# Layer resolving numerical scheme for singularly perturbed parabolic convection-diffusion problem with an interior layer

**DOI:** 10.1016/j.mex.2022.101953

**Published:** 2022-12-02

**Authors:** Gemadi Roba Kusi, Aknaw Hailemariam Habte, Tesfaye Aga Bullo

**Affiliations:** Department of Mathematics, College of Natural Science, Jimma University, Jimma, Ethiopia

**Keywords:** Singularly perturbed, Parabolic problems, Interior layer, Layer resolving, Accurate solution, Layer resolving numerical scheme

## Abstract

Singularly perturbed parabolic convection-diffusion problem with interior layer is a type of singularly perturbed boundary value problems which have sign change properties in the coefficient function of the convection term. This paper introduces a layer resolving numerical scheme for solving the numerical solution of the singularly perturbed parabolic convection-diffusion problem exhibiting interior layer due to the convection coefficient. The scheme is formulated by discretizing the temporal variable on uniform mesh and discretize the spatial one on piecewise uniform mesh of the Shishkin mesh type. The resulting scheme is shown to be almost first order convergent. Theoretical investigations are confirmed by numerical experiments. Moreover, the present scheme is:

• Stable,
• Consistent and
• Gives more accurate solution than existing methods in the literature.

## Graphical abstract

**Figure:**
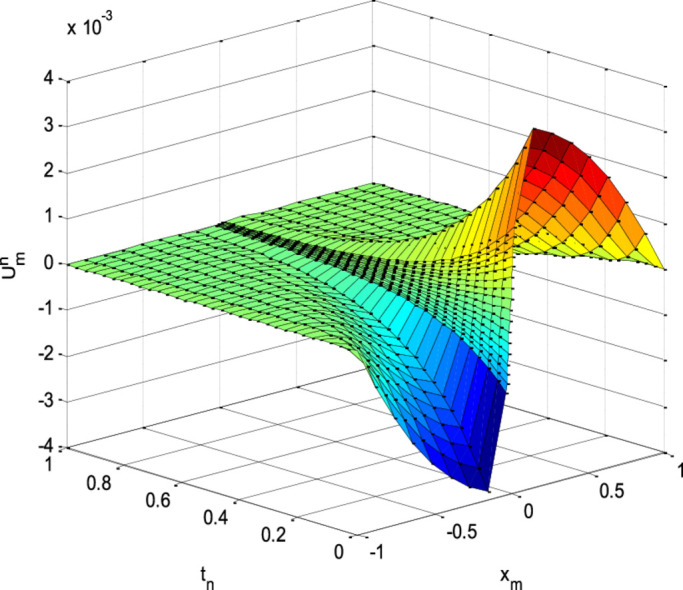

Specifications Table

**Table:** 

Subject area: Mathematics
More specific subject area: Numerical analysis (on the singularly perturbed problems)
Method name: Layer resolving numerical scheme
Name and reference of original method: Bullo, T. A., Degla, G. A., & Duressa, G. F. (2021). Fitted mesh method for singularly perturbed parabolic problems with an interior layer. *Mathematics and Computers in Simulation*, 193, 371–384.
Resource availability: ORCID: 0000-0001-6766-4803

## Introduction

Singularly perturbed parabolic differential equations are parabolic partial differential equations whose highest order derivative is multiplied by a small positive parameter. Parabolic convection-diffusion equations are time-dependent partial differential equations that arise in the physical phenomena where energy, particles, or mass transport flow in any physical system occurs due to both convection and diffusion processes. If one body tries to solve the problem under consideration by using the standard numerical methods, then inaccurate solutions are obtained. Thus, to get accurate numerical solutions, it will be necessary to formulate a robust numerical method that can handle the considered problems. Therefore, the main objective of this study is to develop and analyze robust numerical methods and enhance the more accurate numerical solution for singularly perturbed parabolic problems with an interior layer.

The solution behavior of singularly perturbed convection-diffusion problems highly depends on the nature of the convection coefficient, whether it is vanishing at some points of the domain of the problem or not. Those problems, in which the convection coefficient vanishes at some points of the solution domain, are called singularly perturbed turning point problems, and zeros of the convection coefficient are said to be turning points [1,2]. Turning points appear in various circumstances in science and engineering, particularly in fluid mechanics. They appear at the points of separation of turbulent boundary layers since the tangential velocity vanishes and changes signs at such points. Also, study of the propagation of light in a nonhomogeneous medium as an application of Maxwell's equations leads to the turning point problems, [3]. Hence, the interior layer occurs in the solution of singularly perturbed parabolic problems if the coefficient of convection term and/or the source functions are not sufficiently smooth. In this work, we focus on a singularly perturbed parabolic problem with an interior layer, which happened when the convection coefficient vanishes within the solution domain and its sign changed.

In general, numerical treatment of singularly perturbed parabolic problems is difficult because of the presence of boundary and/or interior layers in its solution. Particularly, classical finite difference or finite element methods fail to yield satisfactory numerical results on uniform meshes and to obtain stability concerning the perturbation parameter, [3], [4], [5], [6], [7]. The layer resolving numerical methods for singularly perturbed problems are widely classified into the fitted operator and fitted mesh methods. In fitted operator methods, exponential fitting parameters will be used to control the rapid growth or decay of the numerical solution in layer regions. Whereas, fitted mesh methods use nonuniform meshes, which will be fine in layer regions and coarse outside the layer regions, [6], [7], [8], [9], [10], [11]. Thus, many layers resolve robust numerical methods developed for different types of singularly perturbed parabolic problems. For the detailed types of a singularly perturbed family of parabolic problems and the developed numerical methods, one can refer to the literature in [12], [13], [14], [15], [16], [17], [18], [19], [20] in addition to the above references.

These referred articles may help us just to get prior knowledge about the nature of the solution of these families of problems and where and why the existing methods in problematic to work. Further, it is a recent and active research area in engineering and applied science. Though many classical numerical methods such as finite difference methods, finite element methods, and finite volume methods have been developed so far, most of them fail to give a more accurate solution, [21,22]. This difficulty is due to the presence interior layer where the solutions vary rapidly and behave smoothly away from the layer. Owing to this, classical numerical methods cannot give a more accurate solution for singularly perturbed parabolic problems; researchers provide attention to formulate methods that may give a more accurate solution.

Moreover, we have been observed that from different presented methods to solve singularly perturbed parabolic problems, the family of fitted operator methods is not applicable for parabolic problems with an interior layer. This is due to the occurrences of interior layers and behaviors of fitted operator methods. Hence, recently, a few numerical schemes [12,13] are proposed to solve singularly perturbed parabolic convection-diffusion problems with an interior layer. However, the obtained solution is yet not satisfactory with the corresponding rate of convergence. Thus, it is necessary to formulate and analyze a higher-order robust numerical method that is uniformly convergent and gives more accurate numerical solutions for the mentioned problems. Therefore, the main aim of this subproject is to answer the questions raised related to the accuracy of the solution, and stability and consistency of the method for the singularly perturbed parabolic problems with an interior layer.

## Statement of the problem

Singularly perturbed parabolic convection-diffusion problems have different types depending on the kind of layers (boundary and/or interior layers). In this work, we proposed a robust numerical scheme for solving the singularly perturbed parabolic convection-diffusion problem with an interior layer defined by:

(1) $$\varepsilon \frac{\partial^{2}u}{\partial x^{2}}+a\left(x,t\right)\frac{\partial u}{\partial x}-b\left(x,t\right)u\left(x,t\right)-c\left(x,t\right)\frac{\partial u}{\partial t}=f\left(x,t\right),\;\;\;\;\forall \left(x,t\right)\in D,$$

subject to the initial and boundary conditions:

(2) $$\begin{array}{c} u\left(x,0\right)=u_{0}\left(x\right),\;\;\;\;\forall x\in \left[-1,\;1\right],\\ u(-1,t)=\alpha ,\;\;\;\;\forall t\in [0,T],\\ u(1,t)=\gamma ,\;\;\;\;\forall t\in [0,T], \end{array}$$

where the solution domain is D≔(−1,1)×(0,T] with positive constant *T* and ε is the perturbation parameter. α and γ are given real numbers. Assume that, functions a(x,t), b(x,t),c(x,t), f(x,t) and $u_{0}\left(x\right)$ are the smooth function to guarantee the uniqueness of u(x,t). Also, Eqs. (1) and (2) is called the singularly perturbed initial boundary value problem due to the existence of the singular perturbation parameter ε as a coefficient of higher order derivative. Further, assume that the inequality$b\left(x,t\right)\geq \beta \geq 0,\;\;\;\forall \left(x,t\right)\in \bar{D}$, ($\bar{D}$ closed solution region), [12,13]. Moreover, it is assumed that the problem has one turning point at x=0, and satisfied the conditions:

(3) $$\left\{ \begin{array}{c} a(0,t)=0,\\ \frac{\partial a(0,t)}{\partial x}>0,\\ a(x,t)<0,\;\;\;\;\;\forall (x,t)\in [-1,\;0)\times [0,T],\\ a(x,t)>0,\;\;\;\;\;\forall (x,t)\in (0,\;1)\times [0,T]. \end{array}\right.$$

The conditions provided in Eq. (3) are used to indicate or guarantees that the layer regions located around the point x=0. Further, Eq. (3) shows that the nature of the problem is reaction-diffusion rather than convection-diffusion at the point x=0 due to absence of the convection term. The last two conditions in Eq. (3) used to indicate the position and type of layer in the considered problem which is at the middle of the spatial domain and an interior layer type.

As a result of the classical numerical methods cannot give a more accurate solution for singularly perturbed parabolic problems, researchers provide attention to formulate methods that may give a more accurate solution. Hence, various numerical schemes are proposed to solve families of these problems, but the obtained solution yet not satisfactory. Thus, due to this causes as the main motivation, it is necessary to develop a robust numerical method that produces a more accurate numerical solution. Thus, the main objective of this work is to provide a robust numerical scheme that produces a more accurate solution for singularly perturbed parabolic convection-diffusion problem with an interior layer.

## Formulation of the numerical scheme

To formulate the scheme, we first discretize the temporal variable on uniform mesh and then discretize the spatial one on piecewise uniform Shishkin mesh consecutively. Hence, the partition of time interval [0,T] with uniform step size *k* is given by:

(4) $$t_{n}=nk,\;\;\;0\leq n\leq N,\;\;\;\;k=\frac{T}{N}.$$

Using Taylor's series expansion about the point $\left(x,t_{n}\right)$, we have

(5) $$u\left(x,t_{n-1}\right)=u\left(x,t_{n}\right)-k\frac{\partial u}{\partial t}\left(x,t_{n}\right)+\frac{k^{2}}{2!}\frac{\partial^{2}u}{\partial t^{2}}\left(x,t_{n}\right)-\frac{k^{3}}{3!}\frac{\partial^{3}u}{\partial t^{3}}\left(x,t_{n}\right)+...$$

From this Eq. (5), we obtain:

(6) $$\frac{\partial u}{\partial t}\left(x,t_{n}\right)=\frac{u\left(x,t_{n}\right)-u\left(x,t_{n-1}\right)}{k}+\tau_{1}$$

where $\tau_{1}=-\frac{k}{2}\frac{\partial^{2}u}{\partial t^{2}}\left(x,t_{n}\right)\equiv O\left(k\right)$

Note that, the error estimate of time discretization is bounded and given by

(7) $${∥E_{n}∥}_{\infty }\leq Ck$$

where $C=\frac{1}{2}{∥\frac{\partial^{2}u}{\partial t^{2}}\left(x,t_{n}\right)∥}_{\infty },\;\;\;\forall n=1,\;\;2,\;...N$, is a constant independent of ε and *k*.

Considering at the nodal point $\left(x,t_{n}\right)$ and substituting Eq. (6) into Eq. (1) yields:

(8) $$\varepsilon \frac{\partial^{2}u}{\partial x^{2}}\left(x,t_{n}\right)+a\left(x,t_{n}\right)\frac{\partial u}{\partial x}\left(x,t_{n}\right)-\left[b\left(x,t_{n}\right)+\frac{c(x,\;t_{n})}{k}\right]u\left(x,t_{n}\right)=f\left(x,t_{n}\right)-\frac{c(x,\;t_{n})}{k}u\left(x,t_{n-1}\right)$$

This indicates that the semi discretize approximation $u(x,t_{n})$ to the exact solution u(x,t)of the differential equation in Eq. (1) at the time levels$t_{n}=nk$.

Now, consider the solution to Eq. (11) has large gradients in a narrow region nearx=d, for dis half of the spatial domain, then the mesh in this region will be fine and coarse everywhere else. Let Mbe a positive integer such thatM≥8. With this in mind, the transition parameter τis chosen to be:

(9) $$\tau =\min\left\{\frac{1}{4},\;2\varepsilon \ln\left(M\right)\right\},$$

where τis a positive constant . Hence, the sub-intervals[−1,−τ], [−τ,τ]and[τ,1] of the domain [−1,1]are subdivided uniformly to contain $\frac{M}{4},\;\frac{M}{2}$and $\frac{M}{4}$mesh elements respectively. While the sub-intervals$\left[0,\;\;\frac{1}{2}-\tau \right]$, $\left[\frac{1}{2}-\tau ,\;\;\frac{1}{2}\right]$$\left[\frac{1}{2},\;\;\frac{1}{2}+\tau \right]$and$\left[\frac{1}{2}+\tau ,\;\;1\right]$ of the domain [0,1]are subdivided uniformly to contain $\frac{M}{4}$mesh elements each. More specifically, the partition of the interval $\left[x_{l},\;x_{r}\right]=\left[-1,\;\;1\right]$ or $\left[x_{l},\;x_{r}\right]=\left[0,\;\;1\right]$is:

$$x_{m}=x_{l}+mh_{m},\;\;\;\;\;\;m=1,2,\;\;...\;\;,M-1,\;\;\;\;\text{for}\;\;\;x_{0}=x_{l}\;\;\text{and}\;\;x_{M}=x_{r}$$

The mesh spacing $h_{m}=x_{m}-x_{m-1}$is given by

(10) $$h_{m}=\left\{ \begin{array}{c} \frac{4(1-\tau )}{M}\;if\;m=1,2,\ldots ,\frac{M}{4},\\ \frac{4\tau }{M},\;\text{if}\;\frac{M}{4}+1,\frac{M}{4}+2,..,\frac{3M}{4},\text{for}\;\;x\in \left[-1,1\right].\\ \frac{4(1-\tau )}{M}\;\text{if}\;m=\frac{3M}{4}+1,...,M. \end{array}\right.$$

(11) $$h_{m}=\left\{ \begin{array}{c} 4\left(\frac{1}{2}-\tau \right),\text{if}\;m=1,2.\ldots \frac{M}{4},\\ \frac{4\tau }{M},\text{if}\;\frac{M}{4}+1,\frac{M}{4}+2,\ldots ,\frac{3M}{4},\text{for}\;x\in \left[0,1\right].\\ \frac{4\left(1-\frac{1}{2}-\tau \right)}{M},\text{if}\;\frac{3M}{4}+1,\ldots ,M. \end{array}\right.$$

We represent this mesh by $D_{M}^{N}$ and for the rest of the paper, any function F(x,t) adopt the notation $F\left(x_{m},t_{n}\right)=F_{m}^{n}$. Thus, the discretize form of Eq. (8) on $D_{M}^{N}$ is formulated depending on the finite difference approximations of the operators:

(12) $$\left\{ \begin{array}{c} \varepsilon \delta_{x}^{2}U_{m}^{n}+a_{m}^{n}\delta_{x}^{{}^{-}}U_{m}^{n}-\left[b_{m}^{n}+\frac{c_{m}^{n}}{k}\right]U_{m}^{n}=f_{m}^{n}-\frac{c_{m}^{n}}{k}U_{m}^{n-1},\;\;\;m=1,2,...\;,\;\frac{M}{2},\\ \varepsilon \delta_{x}^{2}U_{m}^{n}+a_{m}^{n}\delta_{x}^{{}^{+}}U_{m}^{n}-\left[b_{m}^{n}+\frac{c_{m}^{n}}{k}\right]U_{m}^{n}=f_{m}^{n}-\frac{c_{m}^{n}}{k}U_{m}^{n-1},\;\;\;m=\frac{M}{2}+1,\;\;...\;\;M-1, \end{array}\right.$$

where $\delta_{x}^{2}U_{m}^{n}=\frac{2}{h_{m}+h_{m+1}}\left(\delta_{x}^{+}U_{m}^{n}-\delta_{x}^{{}^{-}}U_{m}^{n}\right),\;\;\;\;\;\;\;\;\delta_{x}^{+}U_{m}^{n}=\frac{U_{m+1}^{n}-U_{m}^{n}}{h_{m+1}},\;\;\;\;\;\;\;\;\;\;\delta_{x}^{{}^{-}}U_{m}^{n}=\frac{U_{m}^{n}-U_{m-1}^{n}}{h_{m}}.$

This yields the finite difference approximation for the problem under consideration as:

(13) $$E_{m}U_{m-1}^{n}+F_{m}U_{m}^{n}+G_{m}U_{m+1}^{n}=H_{m}^{n},$$

for $m=1,2,...\;,\;\frac{M}{2}$, $E_{m}=\frac{2\varepsilon }{h_{m}\left(h_{m}+h_{m+1}\right)}-\frac{a_{m}^{n}}{h_{m}}$, $F_{m}=\frac{-2\varepsilon }{h_{m}h_{m+1}}+\frac{a_{m}^{n}}{h_{m}}-b_{m}^{n}-\frac{1}{k}c_{m}^{n}$, $G_{m}=\frac{2\varepsilon }{h_{m}\left(h_{m}+h_{m+1}\right)}$ and for $m=\frac{M}{2}+1,\;\;...\;\;M-1$ and ∀n, we have:

$$E_{m}=\frac{2\varepsilon }{h_{m}\left(h_{m}+h_{m+1}\right)},F_{m}=\frac{-2\varepsilon }{h_{m}h_{m+1}}-\frac{a_{m}^{n}}{h_{m+1}}-b_{m}^{n}-\frac{1}{k}c_{m}^{n},G_{m}=\frac{2\varepsilon }{h_{m}\left(h_{m}+h_{m+1}\right)}+\frac{a_{m}^{n}}{h_{m+1}},H_{m}^{n}=f_{m}^{n}-\frac{c_{m}^{n}}{k}U_{m}^{n-1},\;\;\;\forall \left(m,n\right)$$

## Stability of the method

To investigate the stability estimate for the formulated method, the Von Neumann stability technique is applied to investigate the stability of Eq. (13), by assuming that the solution of this equation at the grid point $\left(x_{m},\;\;t_{n}\right)$ is given by:

(14) $$U_{m}^{n}=\xi^{n}e^{im\theta },$$

where $i=\sqrt{-1}$, with θ is the real number and ξ denotes the amplitude factor. Substituting Eq. (14) into the homogeneous part of Eq. (13) and then solve ξ given as:

$$\xi =\frac{-\frac{c_{m}^{n}}{k}}{\left[\frac{2\varepsilon }{h_{m}\left(h_{m}+h_{m+1}\right)}-\frac{a_{m}^{n}}{h_{m}}\right]e^{-i\theta }+\frac{-2\varepsilon }{h_{m}h_{m+1}}+\frac{a_{m}^{n}}{h_{m}}-b_{m}^{n}-\frac{1}{k}c_{m}^{n}+\;\frac{2\varepsilon }{h_{m}\left(h_{m}+h_{m+1}\right)}e^{i\theta }}$$

The stability condition |ξ|≤1 satisfied as:

$$\left|\xi \right|=\left|\frac{-\frac{c_{m}^{n}}{k}}{\left[\frac{2\varepsilon }{h_{m}\left(h_{m}+h_{m+1}\right)}-\frac{a_{m}^{n}}{h_{m}}\right]e^{-i\theta }+\frac{-2\varepsilon }{h_{m}h_{m+1}}+\frac{a_{m}^{n}}{h_{m}}-b_{m}^{n}-\frac{1}{k}c_{m}^{n}+\;\frac{2\varepsilon }{h_{m}\left(h_{m}+h_{m+1}\right)}e^{i\theta }}\right|\leq 1$$

From the definition problem within the conditions in Eq. (3), we have:

$$\min_{\forall (x,t)}\;\left|a(x,t)\right|\;=0,\;\;\;\;b\left(x,t\right)\geq b_{0}\geq 0.$$

This condition also works for the discrete one and hence|ξ|≤1, which implies the developed finite difference method in Eq. (13) is unconditionally stable.

## Truncation error

For the first half parts the spatial domain, the truncation error *T* between the operators on the exact solution $u(x_{m},\;t_{n})$and approximate solution $U_{m}^{n}$ is given by:

(15) $$T=\left(\varepsilon \frac{\partial^{2}u}{\partial x^{2}}+a\frac{\partial u}{\partial x}-bu-c\frac{\partial u}{\partial t}\right)\left(x_{m},t_{n}\right)-[\varepsilon \delta_{x}^{2}U_{m}^{n}+a_{m}^{n}\delta_{x}^{+}U_{m}^{n}-b_{m}^{n}U_{m}^{n}-c_{m}^{n}\frac{U_{m}^{n}-U_{m}^{n-1}}{k}$$

Using Taylor's series expansions, we have:

(16) $$\delta_{x}^{-}U_{m}^{n}\approx \frac{\partial U_{m}^{n}}{\partial x}-\frac{h_{m}}{2!}\frac{\partial^{2}U_{m}^{n}}{\partial x^{2}}+\frac{h_{m}^{2}}{3!}\frac{\partial^{3}U_{m}^{n}}{\partial x^{3}}+...$$

(17) $$\delta_{x}^{+}U_{m}^{n}\approx \frac{\partial U_{m}^{n}}{\partial x}+\frac{h_{m+1}}{2!}\frac{\partial^{2}U_{m}^{n}}{\partial x^{2}}+\frac{h_{m+1}^{2}}{3!}\frac{\partial^{3}U_{m}^{n}}{\partial x^{3}}+...$$

(18) $$\delta_{x}^{2}U_{m}^{n}=\frac{2}{h_{m}+h_{m+1}}\left(\delta_{x}^{+}U_{m}^{n}-\delta_{x}^{-}U_{m}^{n}\right)\;\;\approx \;\;\frac{\partial^{2}U_{m}^{n}}{\partial x^{2}}-\frac{h_{m+1}-h_{m}}{3}\;\frac{\partial^{3}U_{m}^{n}}{\partial x^{3}}\;\;+...$$

Substituting Eqs. (16)–(18) into Eq. (15) yields the estimated truncation error:

(22) $$T=\left(h_{m+1}-h_{m}\right)C_{1}+C_{2}k,$$

where $C_{1}=\frac{1}{4}{∥-(a_{m}^{n+1}\frac{\partial^{2}U_{m}^{n+1}}{\partial x^{2}}+a_{m}^{n}\frac{\partial^{2}U_{m}^{n}}{\partial x^{2}})∥}_{\infty }$and $C_{2}=\frac{1}{2}{∥\frac{\partial^{2}U_{m}^{n}}{\partial t^{2}}∥}_{\infty }$are constants.

Consideringτ=2εln(M), and $h_{m+1}-h_{m}\leq \frac{4\tau }{M}=\frac{8\varepsilon \ln(M)}{M}$. Also, using the inequality $\varepsilon \leq \frac{4}{M}$we have $h_{m+1}-h_{m}\leq \frac{32\ln(M)}{M^{2}}\leq M^{-1}\ln\left(M\right)$. Therefore, for the exact solution $u(x_{m},t_{n})$ and the approximate solutions $U_{m}^{n}$, we get:

$$\left|u\left(x_{m},t_{n}\right)-U_{m}^{n}\right|\leq C\left(M^{-1}\ln\left(M\right)+k\right),$$

where *C* is independent of perturbation parameter and mesh lengths.

Therefore, the described scheme is almost first-order convergent. A finite difference method is consistent if the limit of truncation error is equal to zero as the mesh size goes to zero. Thus, using this consistency and stability criteria, the proposed method is convergent by Lax's equivalence theorem.

## Numerical illustrations

In this section, two test examples are given for which numerical results are computed to demonstrate the effectiveness of the present method. The maximum absolute errors are calculated by using the exact solution. The solution in the examples has a turning point at x=0 andx=0.5 for Examples 1 and 2, respectively, which gives rise to interior layer. For fixed perturbation parameterε, the maximum absolute errors at all mesh points evaluated using the formulas

$$E_{M}^{N}=\max_{0\leq m\leq M,\;0\leq n\leq N}\left|u_{m}^{n}-U_{m}^{n}\right|$$

where $u_{m}^{n}$ and $U_{m}^{n}$are the exact and approximate solutions evaluated at the nodal points$\left(x_{m},t_{n}\right)$.

Example 1 Consider the singularly perturbed turning point problemwith $a\left(x,t\right)=2x\left[1+\sqrt{\varepsilon t^{2}}\right],b\left(x,t\right)=2\left(2+xt\right)\;\;\;\text{and}\;\;c\left(x,t\right)=1$. This problem has an interior layer of width O(ε), and the exact solution is not available.

Example 2 Consider the singularly perturbed turning point problem

$$\left\{ \begin{array}{c} \varepsilon \frac{\partial^{2}u}{\partial x^{2}}+a\left(x,t\right)\frac{\partial u}{\partial x}-b\left(x,t\right)u-c\left(x,t\right)\frac{\partial u}{\partial t}=f\left(x,t\right),\;\;x\in \left(0,1\right),\;\;t\in \left[0,1\right],\\ u\left(0,0\right)=\varepsilon \tanh\left(\frac{1}{2\varepsilon }\right)-\;{\left(\varepsilon \right)}^{\frac{2}{3}},\\ u\left(1,0\right)=\varepsilon \tanh\left(-\frac{1}{2\varepsilon }\right)-\;{\left(\varepsilon \right)}^{\frac{2}{3}}, \end{array}\right.$$

where $a\left(x,t\right)=2\left(2x-1\right)\left(1+t^{2}\right),\;\;\;\;\;b\left(x,t\right)=2\left(1+xt\right),\;\;\;\;c\left(x,t\right)=\left(1+x^{2}\right)\exp\left(-t\right)$. The exact solution is $u\left(x,t\right)=\varepsilon \exp\left(-\frac{t}{\varepsilon }\right)\tanh\left(\frac{0.5-x}{\varepsilon }\right)-\;{\left(\varepsilon \right)}^{\frac{2}{3}}\exp\left(-xt\right)$ and f(x,t)is obtained after substituting the exact solution. This problem has an interior layer of widthO(ε).

## Discussions and conclusion

**Fig. 1:**
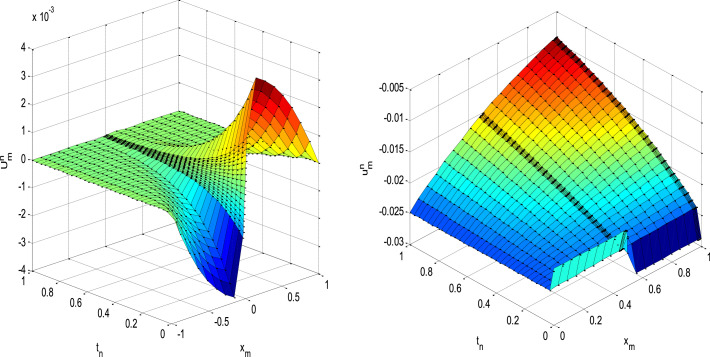
Surface plot of numerical solutions when $\varepsilon =2^{-10},\;\;N=M=32$ for Examples 1 and 2, respectively.

The present method is layer resolving numerical method based on type of Shishkin meshes for solving singularly perturbed parabolic problems with an interior layer. We have recognized the stability and consistency of the formulated method to guarantee its convergence. The main originality of the proposed method explain and investigated in terms the obtained more accurate solutions as our original contributions. The obtained results in Tables 1 and 2 (maximum absolute errors) shows that the error has monotonically decreasing behavior with increasing number of mesh intervals *N* and *M*, which agree convergence of proposed scheme. Comparison of numerical results in Tables shows that, the present scheme gives more accurate results than the scheme given in results in [12,13]. These investigations provide the evidences for the strangeness of the proposed method. Fig. 1, surface plot numerical solutions to visualize the physical understanding of the solution which involves an interior layer and places of the layer clearly. Also, the numerical simulations both in tabular and figures form, indicates the properties and interpretation of numerical solution to support the theoretical descriptions contain about parabolic problems with an interior layer. Further, the method is stable, convergent and gives more accurate solution than some existing methods in literature. Therefore, the presented methods is more appropriate numerical method to gives a more accurate solution which mentioned as the novelty of the paper for the problem under consideration. Furthermore, any interested researcher can apply the proposed method to solve the singularly perturbed parabolic problems with an interior layer due to discontinuity of coefficient or source terms as future work.

**Table 1:** Comparison of maximum absolute errors for Example 1.

ε↓M=N→	16	32	64	128	256
Present Method					
$10^{-3}$	3.9135e-03	2.0772e-03	1.0672e-03	6.3992e-04	3.1918e-04
$10^{-4}$	4.1835e-03	1.8520e-03	8.5472e-04	3.6339e-04	2.4162e-04
$10^{-5}$	4.2993e-03	1.9238e-03	9.1167e-04	4.3817e-04	2.3043e-04
$10^{-6}$	4.3382e-03	1.9462e-03	9.2537e-04	4.5092e-04	2.3931e-04
$10^{-7}$	4.3507e-03	1.9532e-03	9.2908e-04	4.5322e-04	2.4020e-04
$10^{-8}$	4.3547e-03	1.9555e-03	9.3018e-04	4.5376e-04	2.4029e-04
⋮	⋮	⋮	⋮	⋮	⋮
$10^{-12}$	4.3565e-03	1.9565e-03	9.3068e-04	4.5397e-04	2.4029e-04
Results in [13]					
$10^{-3}$	2.5684e-02	1.3697e-02	6.5837e-03	2.8425e-03	1.0021e-03
$10^{-4}$	2.6519e-02	1.4578e-02	7.5016e-03	3.7561e-03	1.8370e-03
$10^{-5}$	2.6607e-02	1.4668e-02	7.5923e-03	3.8469e-03	1.9311e-03
$10^{-6}$	2.6618e-02	1.4678e-02	7.6016e-03	3.8561e-03	1.9402e-03
$10^{-7}$	2.6619e-02	1.4679e-02	7.6026e-03	3.8570e-03	1.9411e-03
$10^{-8}$	2.6620e-02	1.4680e-02	7.6027e-03	3.8571e-03	1.9413e-03
⋮	⋮	⋮	⋮	⋮	⋮
$10^{-12}$	2.6620e-02	1.4680e-02	7.6027e-03	3.8571e-03	1.9413e-03

**Table 2:** Comparison of maximum absolute errors for Example 2.

ε↓N/M→	10/16	40/32	160/64	640/128

Present Method				
$10^{-3}$	1.2643e-02	4.7534e-03	2.7500e-03	1.9526e-04
$10^{-4}$	1.2866e-02	3.4955e-03	1.2391e-03	2.5847e-04
$10^{-5}$	1.3496e-02	3.1482e-03	8.8634e-04	3.0376e-04
$10^{-6}$	1.3593e-02	3.0421e-03	8.0257e-04	2.1975e-04
⋮	⋮	⋮	⋮	⋮
$10^{-14}$	1.3614e-02	3.0421e-03	7.7950e-04	1.9957e-04
Results in [12]				
$10^{-3}$	1.07e−01	3.10e−02	8.00e−03	7.32e−03
$10^{-4}$	1.07e−01	3.13e−02	8.21e−03	2.07e−03
$10^{-5}$	1.07e−01	3.13e−02	8.21e−03	2.08e−03
$10^{-6}$	1.07e−01	3.13e−02	8.21e−03	2.08e−03
⋮	⋮	⋮	⋮	⋮
$10^{-14}$	1.07e−01	3.13e−02	8.21e−03	2.08e−03

## Declaration of Competing Interest

The authors declare that they have no known competing financial interests or personal relationships that could have appeared to influence the work reported in this paper.

## Data Availability

The data that has been used is confidential.
